# Supplementary material for: What Do Young Adults Think about the Safety of Over-the-Counter Analgesics? Findings from a Cross-Sectional Survey
Source: Pharmacy (Basel). 2021 Mar 5;9(1):54. doi: 10.3390/pharmacy9010054 (PMC8005976; doi:10.3390/pharmacy9010054)
Supplement: Supplementary file 1 [file pharmacy-09-00054-s001.pdf]

Young adults and over-the-counter pain medicines (School Leavers)

Welcome to the Survey!

What is this research about?

- How young adults take over-the-counter pain medicines, and what influences them.
- Over-the-counter pain medicines are available from a pharmacy, supermarket, or petrol station, and are able to be purchased without a prescription. Some common brands include Panadol (paracetamol), Nurofen/Advil (ibuprofen), Voltaren (diclofenac) and Naprogesic (naproxen).

Who are we looking for?

- Young adults aged 17 to 25

What you will be asked to do:

- Complete a short 5-10 minute web-based survey on the iPad provided or your own device. All information collected is confidential.

Do I have to participate?

- Participation in this survey is completely voluntary, and you are under no obligation to participate if you do not wish to.
- You are able to withdraw your answers at any point prior to survey completion.
- After survey completion we are unable to withdraw any participants as we cannot identify your response.

Confidentiality

- All of your answers are de-identified and kept completely confidential through the web-based survey program, and no personal information will be collected.

- The results will only be accessed by members of the research team, and any results collected will be kept secure, and disposed of appropriately.

Research involves access to, collection or generation of identified personal information, and there is no plan to disclose identified information to third parties.
The research is strictly not in relation to the Safer Schoolies Gold Coast Response and is purely in relation to young adults and over-the-counter pain medication.

The conduct of this research involves the collection, access and/or use of your identified personal information. The information collected is confidential and will not be disclosed to third parties without your consent, except to meet government, legal or other regulatory authority requirements. A de-identified copy of this data may be used for other research purposes. However, your anonymity will at all times be safeguarded. For further information consult the University's Privacy Plan at http://www.griffith.edu.au/about-griffith/governance/plans-publications/griffith-university-privacy-plan or telephone (07) 3735 5585.

A copy of the full information sheet about this survey can be found at:

https://docs.google.com/document/d/1CpMOtOq4-w2w8bzkr2aRL7vZSeHG1jTtjn8A7TgiYp0/edit?usp=sharing

Alternatively, ask your researchers for a hardcopy.

Griffith
UNIVERSITY
Queensland, Australia

Young adults and over-the-counter pain medicines (School Leavers)

What is your age?

Griffith UNIVERSITY Queensland, Australia

Young adults and over-the-counter pain medicines (School Leavers)

What gender do you best identify with?

- ◯ Male
- ◯ Female
- ◯ Non-binary
- ◯ Prefer not to answer
- ◯ Other (please specify)

Griffith UNIVERSITY Queensland, Australia

Young adults and over-the-counter pain medicines (School Leavers)

What is your current postcode?

Griffith UNIVERSITY Queensland, Australia

Young adults and over-the-counter pain medicines (School Leavers)

Did you leave school in 2019?

- ◯ Yes
- ◯ No

Griffith UNIVERSITY Queensland, Australia

Young adults and over-the-counter pain medicines (School Leavers)

What is the highest year of education you have completed?

- ◯ Year 10 or equivalent
- ◯ Year 11 or equivalent
- ◯ Year 12 or equivalent
- ◯ 1st Year Bachelors
- ◯ 2nd Year Bachelors
- ◯ 3rd Year Bachelors
- ◯ 4th Year Bachelors
- ◯ 1st Year Postgraduate
- ◯ 2nd Year Postgraduate
- ◯ 3rd Year Postgraduate
- ◯ 4th Year Postgraduate
- ◯ TAFE certificate
- ◯ Other (please specify)

____

Young adults and over-the-counter pain medicines (School Leavers)

What are your plans for next year?

- ◯ Working
- ◯ Studying
- ◯ Both
- ◯ Unsure
- ◯ Other (please specify)

Griffith UNIVERSITY Queensland, Australia

Young adults and over-the-counter pain medicines (School Leavers)

Work

Please indicate the main field that this relates to from the list below:

Griffith UNIVERSITY Queensland, Australia

Young adults and over-the-counter pain medicines (School Leavers) Study

Please indicate the main field that this relates to from the list below:

Young adults and over-the-counter pain medicines (School Leavers)

Both study and work

Please indicate the main field that your work relates to from the list below:

Please indicate the main field that your study relates to from the list below:

Griffith UNIVERSITY Queensland, Australia

Young adults and over-the-counter pain medicines (School Leavers)

Are you of Aboriginal or Torres Strait Islander origin?

- ◯ Yes - Aboriginal
- ◯ Yes - Torres Strait Islander
- ◯ Yes - Aboriginal and Torres Strait Islander
- ◯ No
- ◯ Prefer not to answer

Young adults and over-the-counter pain medicines (School Leavers)

Do you have any diagnosed long-term health conditions (lasting longer than 6 months) that requires use of prescription medication(s)?

- ◯ Yes
- ◯ No
- ◯ Prefer not to answer

Griffith
UNIVERSITY
Queensland, Australia

Young adults and over-the-counter pain medicines (School Leavers)

Are any of these prescription medication(s) used to manage pain?

- ◯ Yes
- ◯ No
- ◯ I don't know
- ◯ Prefer not to answer

Young adults and over-the-counter pain medicines (School Leavers)

Do one or both parent(s) or caregiver(s) work in healthcare? (e.g. nurse, doctor, pharmacist, physiotherapist etc.)

- ◯ Yes
- ◯ No
- ◯ Prefer not to answer

Young adults and over-the-counter pain medicines (School Leavers)

How frequently do you use medicines for pain?

Never	Rarely	Few times a year	Every Month	Every Week	Every Day
○	○	○	○	○	○

Griffith UNIVERSITY Queensland, Australia

Young adults and over-the-counter pain medicines (School Leavers)

Where was this medicine available from?

- ◯ Prescription
- ◯ Over-the-counter at a pharmacy or supermarket
- ◯ Both prescription and over-the-counter
- ◯ Prefer not to answer
- ◯ I don't know

Griffith UNIVERSITY
Queensland, Australia

Young adults and over-the-counter pain medicines (School Leavers)

Please rank your level of preference from most likely (1) to least likely (6) for the below statement:

When I want information about over-the-counter pain medicines, I go to...

- Parents
- Other family
- Friends
- Doctor
- Pharmacy
- Internet

Please list any other sources that you might get information about pain medicines from.

Young adults and over-the-counter pain medicines (School Leavers)

Please rate your level of agreement for the following statements:

Taking more medicine than stated on the pack gives a better effect

I don't know	Disagree strongly	Disagree somewhat	Neutral	Agree somewhat	Agree strongly
○	○	○	○	○	○

Optional comment

Griffith UNIVERSITY
Queensland, Australia

Young adults and over-the-counter pain medicines (School Leavers)

I think that over-the-counter pain medicines are safe (they cannot hurt me)

I don't know	Disagree strongly	Disagree somewhat	Neutral	Agree somewhat	Agree strongly
○	○	○	○	○	○

Optional comment

Griffith UNIVERSITY
Queensland, Australia

Young adults and over-the-counter pain medicines (School Leavers)

I have shared/would share pain medicines I can buy from the supermarket and over-the-counter at the pharmacy with my friends

I don't know	Disagree strongly	Disagree somewhat	Neutral	Agree somewhat	Agree strongly
○	○	○	○	○	○

Optional comment

Griffith UNIVERSITY Queensland, Australia

Young adults and over-the-counter pain medicines (School Leavers)

I have taken an over-the-counter pain medicine for a reason other than pain (e.g. sleep, anxiety, performance enhancer, swelling etc.)

- ◯ Yes
- ◯ No
- ◯ Prefer not to answer

Griffith UNIVERSITY Queensland, Australia

Young adults and over-the-counter pain medicines (School Leavers)

What pain medicine and for what reason?

Young adults and over-the-counter pain medicines (School Leavers)

If you are going to use a medicine to relieve pain (e.g. headache), what will you consider when choosing the over-the-counter pain medicine to use? (Please tick all that apply)

- ☐ Previous experience
- ☐ Taste/Coating
- ☐ Medicine size/shape
- ☐ Medicine packaging
- ☐ Whatever I have access to
- ☐ I don't take pain medicines
- ☐ Cost
- ☐ Brand
- ☐ Other (please specify)

____

Young adults and over-the-counter pain medicines (School Leavers)

Where do you usually purchase pain medicines from? (Please tick all that apply)

- [ ] Supermarket
- [ ] Pharmacy
- [ ] Petrol station
- [ ] I don't purchase pain medicines
- [ ] Other (please specify)

Griffith UNIVERSITY Queensland, Australia

Young adults and over-the-counter pain medicines (School Leavers)

When you have pain, which of the following statements best describe your actions

- ◯ I will use over-the-counter products like paracetamol (Panadol) or ibuprofen (Nurofen / Advil) at the first sign of pain
- ◯ I try to treat the cause of the pain before self-medicating
- ◯ I try to use other treatments like herbal products before using pain medicines
- ◯ I don't use pain medicines at all
- ◯ Other (please specify)

Griffith
UNIVERSITY
Queensland, Australia

Young adults and over-the-counter pain medicines (School Leavers)

Your participation is greatly appreciated and we would like your views about related research that might follow. Please tick the box below that best matches your wishes.

- ☐ Only use my data for this specific project
- ☐ The research team can use my data for other related research
- ☐ The research team and other researchers may use my data for related research with ethics approval

If you are interested in getting a summary of results, please follow the link below which will open in another window to put down your best contact details:

https://www.surveymonkey.com/r/OTCsummaryrequest

Griffith UNIVERSITY Queensland, Australia

Young adults and over-the-counter pain medicines (School Leavers)

Thank you for participating! Please bring the iPad to one of the researchers to receive your voucher.

Voucher number:
